# Dietary changes during the COVID-19 lockdown in Iranian households: are we witnessing a secular trend? A narrative review

**DOI:** 10.3389/fpubh.2024.1485423

**Published:** 2024-10-25

**Authors:** Bahareh Nikooyeh, Delaram Ghodsi, Maryam Amini, Samira Rabiei, Hamid Rasekhi, Mohammad Esmaeil Motlagh, Tirang R. Neyestani

**Affiliations:** ^1^Department of Nutrition Research, National Nutrition and Food Technology Research Institute and Faculty of Nutrition Sciences and Food Technology, Shahid Beheshti University of Medical Sciences, Tehran, Iran; ^2^Department of Pediatrics, Faculty of Medicine, Ahvaz Jundishapur University of Medical Sciences, Ahvaz, Iran

**Keywords:** COVID-19, lockdown, dietary changes, policy-making, surveillance

## Abstract

**Background:**

The COVID-19 pandemic, which emerged in late 2019, influenced nearly all aspects of human life, including food choices and dietary habits.

**Methodology:**

A web-based search was conducted from March to April 2024 in MEDLINE/PubMed, CINAHL, Embase, and the Cochrane Library for published reports of large-scale surveys of dietary changes during or shortly after the coronavirus pandemic lockdown in Iran. The keywords used were coronavirus OR COVID-19, diet OR nutrition, household, and Iran. Studies that focused on specific areas, subgroups (such as students), or just one city or province were not included. To monitor dietary changes from the years before the lockdown, we used and plotted data from the Household Income and Expenditure Survey (HIES), as provided by the Statistical Center of Iran.

**Results:**

The overall results of the nationwide studies conducted during the lockdown period in Iran revealed that a significant proportion of the households reduced their consumption of milk, yogurt, cheese, red meat, white meat, eggs, and rice/bread. In approximately 47% of the households where weekly consumption of animal protein sources decreased, the consumption of rice and bread increased. Accordingly, 35 and 44% of households reported a decrease in the consumption frequency of fruits and snacks, respectively. Additionally, 21% of those who reduced their fruit consumption completely removed fruits from their food basket. Meanwhile, the use of dietary supplements, especially vitamin D, vitamin C, zinc, and multivitamins, had increased in households, particularly among those with higher incomes. Decreased income was a common reason for all of these dietary changes, which can have major health consequences in the long term.

**Conclusion:**

This review provides evidence that the observed changes had already begun years before the pandemic and presumably have not yet returned to the pre-pandemic situation. Finally, we suggest some strategies for policymakers to overcome the crisis and enhance community the nutrition and health of general population.

## Introduction

The year 2019 did not end happily due to the emergence of a new coronavirus infection (COVID-19) that caused countless cases of morbidity and mortality worldwide (1, 2). COVID-19 quickly became a global concern, as almost no one felt immune to it (3, 4). This fear influenced many aspects of people’s lives, including their food choices and dietary patterns (5–8). On the other hand, authorities implemented lockdowns, and health organizations strongly recommended social distancing. The lockdown had a significant impact on the socioeconomic situation, resulting in decreased income and even job loss for many households (9, 10). Consequently, food choices and dietary habits of a significant proportion of the population changed. However, these dietary changes did vary within and between countries. A study conducted on a sample of the Spanish population revealed that during the pandemic, consumption of unhealthy foods increased while physical activity decreased (11). In a similar study involving 1,097 adults from Poland, almost half of the participants reported increased eating and snacking (43 and 52%, respectively), and approximately one-third reported weight gain, which was associated with decreased consumption of fruits, vegetables, and legumes but an increased intake of meat, dairy products, and fast foods. Approximately 15% of the participants increased their alcohol consumption during lockdown (8). In contrast, an Italian survey of dietary changes during the pandemic revealed greater adherence to the healthy Mediterranean diet among 18- to 30-year-old participants compared to both younger and older participants (12). Overall, these findings suggest both positive and negative impacts of the COVID-19 pandemic on dietary habits worldwide (13). In a study conducted by the U.S. Department of Agriculture (USDA) using the Consumer Expenditure Diary Survey to evaluate food-buying behaviors before and during the early onset of the COVID-19 pandemic, a decrease in the consumption of full-service restaurant meals was observed. This was accompanied by an increase in the purchase of food items from grocery stores, primarily for protein foods, fruits, and vegetables (14).

With the development of COVID-19 vaccines in 2020, an undoubtedly remarkable success in medical history, an estimated 14.4–19.8 million deaths were prevented in 185 countries from 8 December 2020 to 8 December 2021 (15). Although the COVID-19 vaccinations provided an opportunity for human life to return to normal, it left unanswered an important question: Did the dietary changes adopted during the lockdown persist afterward? Some studies have examined these changes following the pandemic. For instance, in Saudi Arabia, approximately 41% of participants in an online survey reported weight gain, while 21% reported weight loss after the COVID-19 pandemic. Both healthy and unhealthy dietary changes, which included increased intake of fruits and vegetables and increased consumption of fast foods and sugary snacks, were reported by approximately one-third of the participants (16). In an attempt to evaluate the long-term effects of the pandemic on dietary habits, a cross-sectional study involving 118 adults residing in the region of Lazio, Italy, found that after the COVID-19 pandemic, they consumed more vegetables, whole grains, and water and ate less out (17). Another online survey involving 1,005 adults conducted in Bahrain found an increase in the consumption of sugary drinks and fast food (18). These post-COVID-19 changes in dietary habits will have health implications.

In this review, we evaluated the overall dietary changes in Iranian households during the COVID-19 lockdown period and compared them with the available data on the trends of food consumption since 2016. Briefly, the aim of this review is to: (i) provide an overview of the overall dietary changes in Iranian households during the lockdown period, (ii) evaluate whether these changes are persistent, and (iii) assess their potential health consequences. Finally, some recommendations are provided for policymakers to address pandemic-induced unhealthy dietary changes and enhance the nutritional status of the general population in Iran.

## Methodology

A thorough literature search on dietary changes during or shortly after the coronavirus pandemic lockdown was conducted using electronic databases, such as MEDLINE/PubMed, CINAHL, Embase, and the Cochrane Library. The keywords used were coronavirus OR COVID-19, diet OR nutrition, household, and Iran. The search focused on English-language articles published between 2020 and 2024. To track dietary changes from years before the lockdown, we used and plotted data from the Household Income and Expenditure Survey (HIES), the Statistical Center of Iran. The exclusion criteria included study protocols alone, meta-analyses, systematic reviews, rapid reviews, and narrative reviews. In addition, studies that focused on a specific area, subgroups (such as students), or cities or provinces were also excluded. The selected literature was chosen according to the predefined inclusion criteria, starting with the title, followed by the abstract, and concluding with the full text. The initial search yielded 79 studies, of which 32 were duplicates and 21 were excluded based on their titles. After screening the abstracts of the remaining 26 studies, 18 were excluded for irrelevance, leaving eight studies for inclusion in the review.

## Results

Table 1 shows the nutrition surveys conducted in Iran during the pandemic. Studies that focused on a specific subpopulation (such as students), a specific area, city, or province were not included. Nevertheless, the majority of the studies had small sample sizes. The largest survey during the lockdown period was carried out using a web-based, electronic, self-administered questionnaire, the protocol for which has been described elsewhere (19). Data obtained from 21,290 households from 4 to 25 April 2020 were analyzed. The majority of respondents reported decreased consumption of major food groups. Specifically, the consumption of milk, yogurt, and cheese decreased in approximately 29, 26, and 7% of the households, respectively (20). Similarly, the consumption frequency of red meat, white meat, eggs, and rice/bread in a week also showed a decrease in 33, 24, 14.2, and 7% of the households, respectively. In approximately 47% of the households where the weekly consumption of animal protein sources decreased, the intake of rice and bread increased (21). Accordingly, 35 and 44% of the respondents reported decreased consumption frequency of fruits and snacks, respectively. Additionally, 21% of those who reduced their fruit consumption completely removed fruits from their food basket (22). Even fast food consumption significantly decreased in approximately 75% of the responding households (23). Interestingly, the use of dietary supplements, especially vitamin D, vitamin C, zinc, and multivitamins, increased in households, particularly among those with higher incomes (24). Decreased income was a common reason for all these dietary changes.

**Table 1:** Nutrition surveys conducted during the COVID-19 pandemic in Iran.

Study*	Methodology	Period of data gathering	Population	Number of participants	Main findings
Mohajeri (55)	Cross-sectional; Electronic food choice questionnaire	April 2019–May 2020	18–66 y Iranian adults	2,540 individuals	Increased consumption of vegetables, fruits, legumes, and nuts after the lockdown
Rabie (23)	Cross-sectional; Electronic self-administered food frequency questionnaire	4–25 April 2020	Iranian households	21,290 households	Decreased consumption of fast food in 74.8% of households
Nikooyeh (21)	Cross-sectional; Electronic self-administered food frequency questionnaire	4–25 April 2020	Iranian households	21,290 households	Decreased weekly consumption of red meat, white meat, eggs, and rice/bread in 33, 24, 14.2, and 7% of the households, respectively
Nikooyeh (20)	Cross-sectional; Electronic self-administered food frequency questionnaire	4–25 April 2020	Iranian households	21,290 households	Decreased weekly consumption of milk, yogurt, and cheese in 29, 26, and 7% of the households, respectively. Decreases in consumption were more pronounced in female-headed households and in food-insecure provinces
Akbarzadeh (56)	Cross-sectional; Online food frequency questionnaire	27^th^ March 27–29^th^ April 2020	Iranian adults aged >18 y	1,553 individuals	Increased consumption of protein-rich foods except for fish and dairy; decreased consumption of fast foods; and increased consumption of fruits, vegetables, and supplements
Ghodsi (24)	Cross-sectional; Electronic self-administered food frequency questionnaire	4–25 April 2020	Iranian households	21,290 households	Nutritional supplements were used in 27% of the households, primarily vitamins D and C; supplement use was less common in low-income households and in food-insecure provinces
Maharat (57)	Cross-sectional; Online questionnaire	During the pandemic	Iranian adults aged >18 y	1,106 individuals	Decreased consumption of milk, yogurt, red meat, fish, and fast foods; increased consumption of whole-grain bread, legumes, nuts, vegetables, and fruits was more pronounced in higher socioeconomic status
Amini (22)	Cross-sectional; Electronic self-administered food frequency questionnaire	4–25 April 2020	Iranian households	21,290 households	Decreased consumption of fruits and snacks in 35 and 44% of the households, respectively. The decreased consumption was more pronounced in low-income households and in food-insecure provinces

*All studies were cross-sectional and descriptive. Studies limited to a specific area, subgroups (such as students), or a specific city or province were not included.

The impact of economic factors on food choices and dietary patterns has long been documented (25). Meanwhile, the coronavirus pandemic, the biggest threat since World War II, caused significant damage to the global economy. Industry, commerce, business, and agriculture were all adversely affected to varying degrees (26). While the process of post-pandemic economic recovery has begun in many countries (27), the consequences may persist as behavioral changes and affect the dietary patterns of the survivors (28).

### 3.1 Will coronavirus pandemic-induced dietary changes in Iran persist?

**Figure 1:**
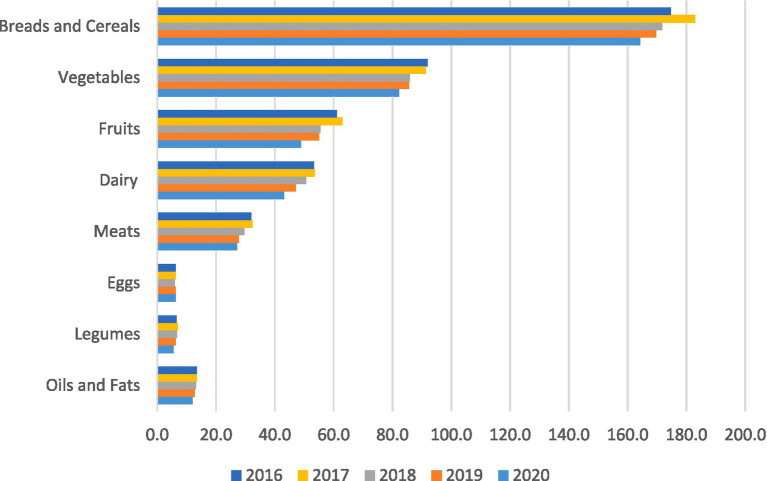
Changes in *per capita* consumption of different food groups among Iranian households (g/d) from 2016 to 2020 (Source: Household Income and Expenditure Survey (HIES), Statistical Center of Iran).

The crucial question is whether these largely unhealthy changes in dietary intake in Iranian households will revert to pre-pandemic levels. Iran’s economy was not immune to the adverse impacts of the pandemic (29). The biggest difference between Iran and many other countries is that Iran’s economy was already seriously suffering from rigid sanctions (30), which led to inflation (31) that was further exacerbated by the pandemic. Unfortunately, inflation did not subside following the pandemic. We believe that these changes had already begun years before the pandemic, but were accelerated during the lockdown period. Figure 1 shows the *per capita* consumption of different food groups from 2016 to 2020, according to the Statistical Center of Iran, which uses food item purchases as a proxy for consumption and provides data up to 2020 (32). Consumption of meat and dairy products, especially milk and yogurt, bread, and cereals has shown a decreasing trend since 2016, three years before the pandemic. Another study also confirmed a declining trend in overall dairy consumption after the COVID-19 pandemic (33). Notably, food purchasing behavior varies among households in different income groups. High-income households spend more on all food groups except hydrogenated oils and animal fats (34). This suggests that the old story repeats itself: low-income households suffer more from the aforementioned changes in dietary intake. Given that the determinants of inflation in Iran have not improved, it is expected that the observed dietary changes will persist.

### What are the possible consequences of the persistence of the observed dietary changes during the lockdown?

The decreased contribution of animal protein food sources due to reduced access and increased reliance on cereals and oils in the household food basket may lead to a diet with low nutrient density, resulting in a combination of overweight and micronutrient deficiencies (35, 36). The quality of the diet may be even poorer when considering the reduced or absent consumption of dairy products (particularly milk and yogurt) and fruits. It is noteworthy that the *per capita* consumption of dairy products in Iran before the pandemic was significantly lower than the global average (~165 g/d *per capita*) (37) and below the recommended intake of three servings a day (38). Decreased consumption of dairy products may lead to a lower intake of high-quality protein, fatty acids, vitamins, and minerals, including bioavailable calcium (39), which predisposes individuals to metabolic bone disease (38, 40). Meanwhile, lower consumption of fruits, below 140 g/d, as evident in Figure 1, indicates a decreased intake of dietary fiber, antioxidants, and health-promoting phytochemicals that have preventive properties against various human disorders, including cancer, gastrointestinal diseases, and cardiovascular diseases (41, 42). Although the use of nutritional supplements increased among Iranian households during the lockdown period, it is unlikely to compensate for potential dietary deficits, especially when considering that supplement use is less common among households with lower socioeconomic status (24). Decreased consumption of fast foods during the lockdown period may be promising (23). However, we do not have any data on the current situation of fast food consumption in Iran. Health literacy has been reported to be the main determinant of fast food consumption among the Iranian population (43), a post-pandemic increase is likely due to the existing barriers to a healthy diet in Iran (44).

The strength of this review is that it provides an overview of the dietary changes in Iranian households during the COVID-19 lockdown period and compares them with the available data since 2016. The latter point is very critical as it shows that these changes were a continuation of a trend that began years ago and presumably continues to this day. Nevertheless, some limitations must be acknowledged. There are no quantitative data on individual or household food consumption during the lockdown, as conducting a consumption survey using classical methodologies, such as 24-h recall, was not possible during this period. Notwithstanding, the data obtained from the nationwide study (19) were reasonably consistent with the data obtained from the Statistical Center of Iran.

Overall, the dietary trends observed in the Iranian population significantly diverge from the recommended dietary guidelines (45, 46). Such non-adherence to healthy dietary guidelines can lead to severe consequences, including increased morbidity and mortality (47).

### What are the potential solutions to this crisis? Recommendations for policymakers

The present article highlights the significant dietary changes observed in Iranian households during the COVID-19 lockdown and raises important questions about the persistence of these changes. Some potential solutions to this crisis and recommendations for policymakers based on the information provided are as follows:

(i) **Social and economic support**

Considering that the most important reason for dietary changes was a decrease in income and economic power, policymakers should focus on providing economic and social support to households, especially those in lower income categories. These measures can include financial aid, food assistance programs, and, above all, employment support for businesses adversely affected. However, it is important to note that these measures can only reduce the effects of the crisis in the short term (48).

(ii) **Nutritional education and awareness**

Implementing educational programs and awareness-raising campaigns to improve nutritional and health literacy is crucial. Many households may have changed their dietary patterns due to economic problems or misconceptions about healthy foods that emerged during the pandemic. Programs that promote nutrition literacy can help individuals make better food choices even during an economic crisis (49).

(iii) **Price controls and subsidies**

Implementing price control programs and providing subsidies for essential food items can help improve conditions. Undoubtedly, subsidized food items should be selected carefully. This can make healthy food options more affordable and accessible to a broader segment of the population (48).

(iv) **Supporting local agriculture**

Paying special attention to local food production can help ensure a stable supply of fresh and nutritious food. This approach is cost-effective in the long term, especially in economically disadvantaged areas (50).

(v) **Public–private partnerships**

Collaborating with private and non-governmental organizations can be effective in improving food security. Aligning the activities of non-governmental and governmental organizations can lead to more comprehensive and sustainable solutions (51).

(vi) **Facilitating healthcare access**

Quick and easy access to healthcare services should be ensured, especially for vulnerable populations. Regular examinations, early dietary counseling, and access to supplements at the primary level of the healthcare system can help reduce micronutrient deficiencies (52).

(vii) **Monitoring and surveillance**

It is critical to establish an efficient and sustainable food and nutrition surveillance (FNS) system to monitor changes in dietary patterns over time and predict future events in a timely manner. This system will allow policymakers to check the effectiveness of interventions and make informed decisions (53).

(viii) **Research and policy evaluation**

Investing in research to better understand the factors affecting dietary changes and the impacts of various interventions can help stakeholders make informed decisions. Regular evaluation of policies will enable policymakers to improve planning and make decisions based on evidence.

(ix) **International collaborations**

Engaging in international collaborations to share best practices and learn from the experiences of other countries facing similar challenges can enhance evidence-based policymaking (54).

There are some limitations to the current study. Almost all studies included in this review used self-administered web-based questionnaires, with no control over who completed the questionnaire. In addition, no quantitative data on food consumption were provided. Nevertheless, data obtained from pre-pandemic dietary surveys, combined with the reported changes in usual food intake, may provide a somewhat clear picture of the dietary situation of Iranian households during the lockdown.

## Conclusion

The COVID-19 pandemic unleashed unique challenges worldwide, influencing every aspect of human life. Fear of the virus, along with lockdowns and economic downturns, caused significant changes in food choices and dietary patterns.

Dietary alterations observed worldwide during and after the lockdown revealed a complex interplay of socioeconomic factors, health behaviors, and regional circumstances. In Iran, a large-scale survey highlighted a worrying decline in the consumption of key food groups, which has been exacerbated by economic challenges.

The present report rightly emphasizes the long-term consequences of sustained dietary changes. A decrease in animal protein sources and an increase in the consumption of cereals and oils might result in a low-nutrient density diet, potentially resulting in overweight and micronutrient deficiencies. Downward trends in dairy and fruit consumption raise concerns about nutritional adequacy, with potential repercussions for bone health and disease prevention.

To navigate this crisis, policymakers must adopt a multi-faceted approach. Economic support, nutritional education, and price controls on essential foods are crucial short-term measures. Long-term strategies include supporting local agriculture, fostering public–private partnerships, and ensuring easy access to healthcare services. Regular monitoring of dietary changes through an FNS system, investing in research, and fostering international collaborations are essential for evidence-based policymaking. The proposed recommendations provide a roadmap for policymakers to address the challenges posed by these changes, aiming for a healthier and more resilient post-pandemic future.
